# Plasma miR-601 and miR-760 Are Novel Biomarkers for the Early Detection of Colorectal Cancer

**DOI:** 10.1371/journal.pone.0044398

**Published:** 2012-09-06

**Authors:** Qifeng Wang, Zhaohui Huang, Shujuan Ni, Xiuying Xiao, Qinghua Xu, Lisha Wang, Dan Huang, Cong Tan, Weiqi Sheng, Xiang Du

**Affiliations:** 1 Department of Pathology, Cancer Hospital, Fudan University, Shanghai, China; 2 Department of Oncology, Shanghai Medical School, Fudan University, Shanghai, China; 3 Institutes of Biomedical Sciences, Fudan University, Shanghai, China; 4 Wuxi Oncology Institute, the 4th Affiliated Hospital of Suzhou University, Wuxi, Jiangsu, China; 5 Fudan University Shanghai Cancer Center - Institute Mérieux Laboratory, Shanghai, China; Baylor University Medical Center, United States of America

## Abstract

**Background:**

Colorectal cancer (CRC) is a major cause of death worldwide. Sensitive, non-invasive diagnostic screen methods are urgently needed to improve its survival rates. Stable circulating microRNA offers unique opportunities for the early diagnosis of several diseases, including cancers. Our aim has been to find new plasma miRNAs that can be used as biomarkers for the detection of CRC.

**Methodology/Principal Findings:**

According to the results of miRNA profiling performed on pooling plasma samples form 10 CRC patients or 10 healthy controls, a panel of miRNAs (hsa-miR-10a, -19a, -22*, -24, -92a, 125a-5p, -141, -150, -188-3p, -192, -210, -221, -224*, -376a, -425*, -495, -572, -601, -720, -760 and hsa-let-7a, -7e) were deregulated in CRC plasma with fold changes >5. After large scale validation by qRT-PCR performed on another 191 independent individuals (90 CRC, 43 advanced adenoma and 58 healthy participants), we found that the levels of plasma miR-601 and miR-760 were significantly decreased in colorectal neoplasia (carcinomas and advanced adenomas) compared with healthy controls. ROC curve analysis showed that plasma miR-601 and miR-760 were of significant diagnostic value for advanced neoplasia. These two miRNAs together yield an AUC of 0.792 with 83.3% sensitivity and 69.1% specificity for separating CRC from normal controls, and yield an AUC of 0.683 with 72.1% sensitivity and 62.1% specificity in discriminating advanced adenomas from normal controls.

**Conclusions/Significance:**

Plasma miR-601 and miR-760 can potentially serve as promising non-invasive biomarkers for the early detection of CRC.

## Introduction

CRC accounts worldwide for 600 thousands deaths per year. In China, CRC remains the 4th most common cause of cancer-related deaths to date [1], [2]. The tumor stage at diagnosis is the most important prognostic factor in CRC, making early detection of crucial importance in reducing mortality and improving compliance rates. Novel biomarkers for early detection are urgently needed. Advanced adenomas are typically defined as adenomas of 1 cm or greater, and those with villous components (tubulovillous or villous) or high-grade dysplasia [3], [4]. Advanced adenomas clearly link up the chain from benign adenoma to advanced CRC. Thus, most CRC screening strategies focus on the detection rate of both CRC and advanced adenomas.

MiRNAs are small non-coding RNAs (18–22 nt in length) that regulate the expression of target genes at the post-transcriptional level by binding to target mRNA. Since their discovery in 1993, altered expressions of miRNAs have been associated with many kinds of tumors, including CRC [5], [6]. Their association with tumor genesis indicates their potential as diagnostic markers and therapeutic targets [7], [8].

Circulating miRNAs are stably detected in plasma/serum and serve as biomarkers for several diseases [9], [10], [11], [12], [13], [14], making them potentially useful non-invasive markers for early diagnosis or in monitoring cancer progression. We previously found that elevated levels of miR-29a and miR-92a in plasma have significant diagnostic value for CRC [15]. To strengthen the diagnostic efficiency of circulating miRNA, it is necessary to identify new targets. In this study, we found that plasma miR-601 and miR-760 concentrations could contribute to early detection of CRC according to the chip profiling and subsequent large scale validation using qRT-PCR.

## Materials and Methods

### Study Population

A group of 10 CRCs (including 5 stage II and 5 stage III patients) and 10 normal controls were first prepared for miRNA profiling (Table S1). Independent 191 age and gender-matched individuals were recruited in for large-scale qRT-PCR validation, including 90 CRC patients (Stage I-IV), 43 advanced adenoma patients and 58 healthy participants (Table S2, S3). Paired tissue samples used for the analysis of selected miRNAs expression were provided by another 19 CRC patients (Table S4). Patients with a previous history of malignant tumors, hereditary non-polyposis CRC or familial adenomatous polyposis were excluded from this study. Blood samples were collected before operation and tissue samples were collected after surgery. Tumor was staged according to the International Union against Cancer (UICC) guidelines. The Clinical Research Ethics Committee of Fudan University Cancer Hospital approved the research protocols and written informed consents were obtained from the participants.

### RNA Isolation

An amount of 5 to 10 milliliters of whole blood were obtained from each participant. The plasma was obtained by centrifugation at 1200 g for 10 min at 4°C. To complete the removal of residual cellular components, plasma samples were re-centrifuged at 12,000 g for a further 10 min at 4°C. The supernatant plasma was frozen as separate aliquots at −80°C until use. This procedure was carried out within 2 h after venipuncture. A volume of 600 µL of thawed plasma was heated at 65°C for 10 min and then incubated at 42°C for 1 h to denature the protein. Total RNA was extracted from the pre-heating plasma using MirVana PARIS Kit (Ambion, USA), eluting it into 60 uL (95°C) RNase-free water. Then plasma RNAs were concentrated in a final volume of 20 µL using an Eppendorf Concentrator Plus 5301(Eppendorf, Germany). For normalization of sample-to-sample variation during RNA isolation and as internal control, same amounts (25 fmol) of synthetic C. elegans miRNA-39 (cel-miR-39) was added into each denatured sample. Total RNA of CRC tissues was extracted using TRIzol reagent(Invitrogen, USA) according to the provided protocol. The concentration of RNA samples was quantified by NanoDrop ND-1000 (Nanodrop, USA).

### MiRNA Profiling

MiRNA profiling was done with a miRCURY LNA™ Universal RT microRNA PCR system (Exiqon, Demark) on 2 pooled plasma samples from 10 CRCs (including 5 stage II and 5 stage III patients) and 10 normal controls. The method is based on universal reverse transcription (RT) followed by quantitative real-time PCR amplification with LNA™ enhanced primers using SYBR Green. Pre-aliquoted LNA™ PCR primers were set in 384-well PCR plates for each reaction per well [16]. A volume of 600 µL of each plasma samples was picked out and uniformly mixed. Total RNA was prepared as described above and an amount of 500 ng was used for next profiling. A total of 742 target miRNAs was detected and fold changes of each miRNA expression were calculated.

### Quantification of miRNAs by qRT-PCR

About 30 ng enriched RNA was reverse transcribed with a TaqMan microRNA Reverse Transcription Kit (Applied Biosystems, USA) in a 10 µL reaction volume. RT products were used as templates for the next PCR process after a 1∶10 dilution. Expression levels of miRNAs were quantified in triplicate by qRT-PCR using human TaqMan MicroRNA Assay Kits (Applied Biosystems, USA) on an ABI 7900 HT qRT-PCR instrument (Applied Biosystems, USA). Both spiked in cel-miR-39 and endogenous miR-16 were used as normalizers for plasma miRNA quantification, while RNU6B for tissue samples.

### MiRNA Stability in Plasma

The same plasma sample, divided into several aliquots, was subjected to a series of treatments, such as incubating at room temperature for different times (1, 2, 4, 8, 16, 24 h) or subjecting them to different numbers of freeze/thaw cycles from storage temperature (−80°C) to room temperature(22°C). To assess the stability of plasma miR-601 and miR-760, Ct values detected by qRT-PCR reflecting concentrations of experiment samples were compared with those undergoing the standard protocols.

### Statistical Analysis

The relative expression of miRNAs was analyzed by the 2^−△△Ct^ method. The Mann-Whitney U test was used to compare the expression of plasma miRNAs between the different groups. Receiver-operating-characteristic (ROC) curves and the area under the ROC curve (AUC) assessed the feasibility of using plasma miRNA as a diagnostic tool for CRC. The Younden index determined the threshold for the plasma miRNA concentrations. The correlation between clinicopathologic features and plasma miRNA levels was determined by Mann-Whitney U test, Student’s t-test or Χ2 test. All tests were 2-sided and a significance level of P<0.05 (95% CI) was considered statistically significant. Statistical analysis relied on SPSS 16.0 software (SPSS Ltd., UK) and graphs were generated with Graphpad Prism 5.0 (Graphpad Software Inc, USA).

## Results

### MiRNA Profiling

After excluding several miRNA targets with Ct values >35 in cancer samples for their extremely low expression, 86 miRNAs were differentially expressed between CRC and control with fold changes >2 (Table S5). Both miR-29a and miR-92a were included in the up-regulated list as we previously reported [15]. Some other deregulated targets, such as has-miR-95, -181b, -200c, -220 and -221, were previously reported in CRC [13], [17]. The raw data of miRNA profiling has been uploaded on the GEO database(GSE38716). In order to narrow the scope of study, a panel of 9 up-regulated miRNAs (hsa-miR-19a, -22*, -24, -92a, -125a-5p, -210, -221, -376a and hsa-let-7e) and a panel of 13 down-regulated miRNAs (hsa-miR-10a, -141, -150, -188-3p, -192, -224*, -425*, -495, -572, -601, -720, -760 and hsa-let-7a) were selected with fold changes >5. In considering our aim of finding novel plasma biomarkers, 5 previous reported targets (hsa-miR-92a, -141, -210, -221 and hsa-let-7a) were removed [13], [17], [18], [19], [20], [21], [22], and the remaining 17 miRNAs were selected for further large-scale qRT-PCR validation.

### Diagnostic Value of miR-601 and miR-760 for CRCs

**Figure 1:**
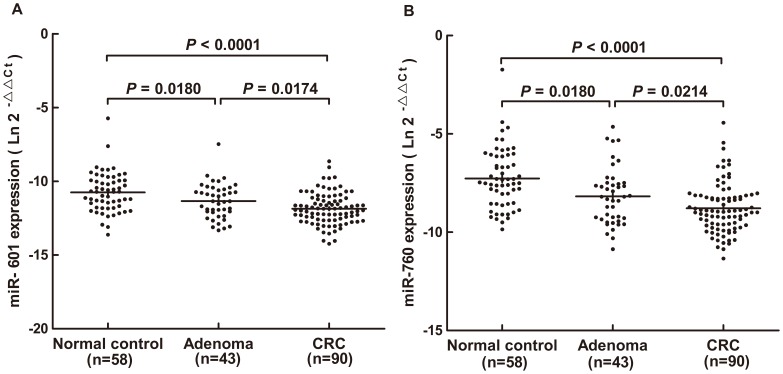
Plasma miR-601 and miR-760 expression. Large-scale validation of miR-601 and miR-760 in plasma samples (n = 191). Scatter plots of plasma levels of miR-601(A) and miR-760(B) in healthy subjects (n = 58), advanced adenomas(n = 43) and CRC patients(n = 90). Expression levels of the miRNAs were normalized to cel-miR-39. The line represents the median value. Mann-Whitney U test was used to determine statistical significance.

**Figure 2:**
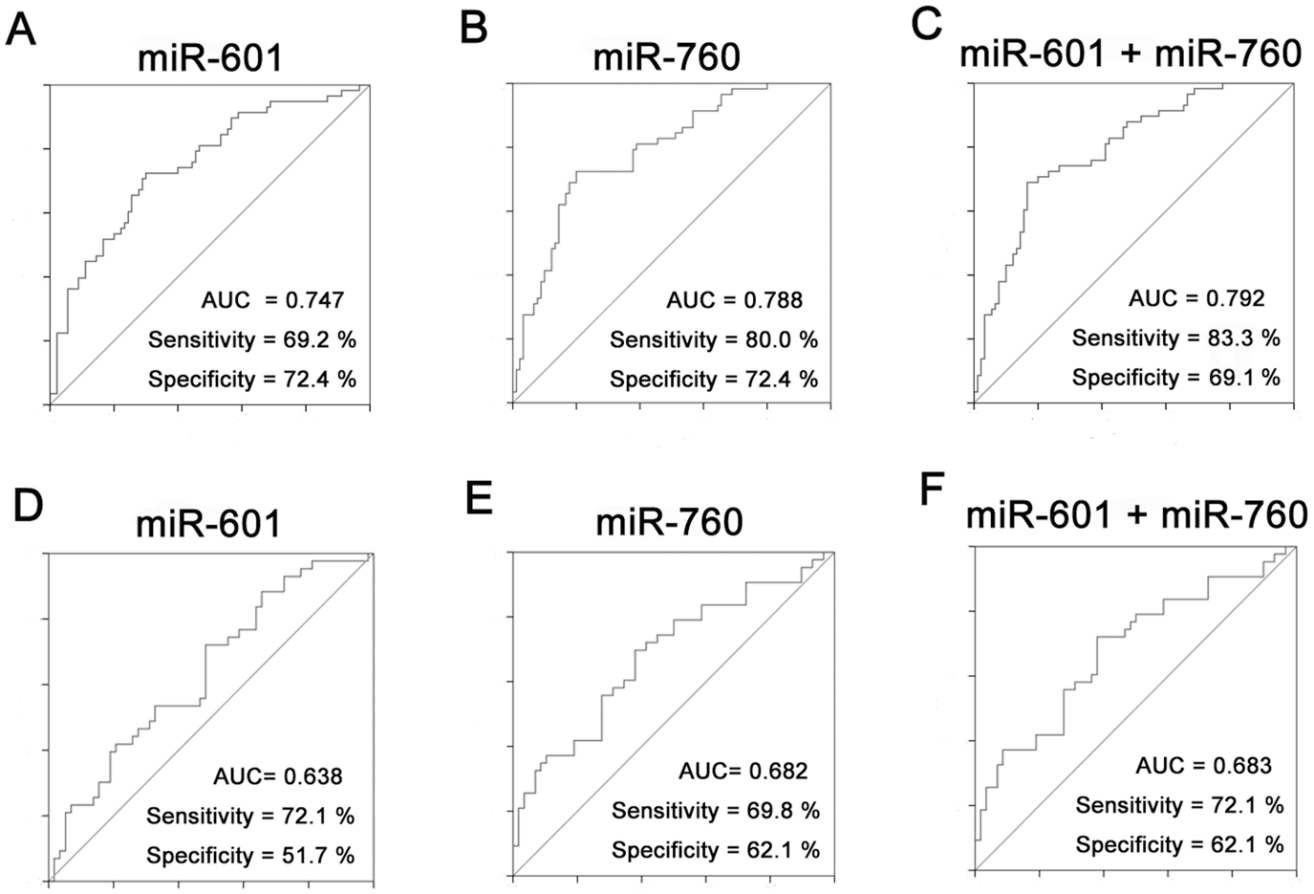
ROC curve analysis of plasma miR-601 and miR-760. (A, B, C) MiR-601 yield an AUC of 0.747(95% CI: 0.666–0.828) with 69.2% sensitivity and 72.4% specificity(cut-off value = −11.50), and miR-760 yield an AUC of 0.788(95% CI: 0.714–0.862) with 80.0% sensitivity and 72.4% specificity(cut-off value = −8.09) for discriminating CRC from normal controls. Combined analysis of miR-601 and miR-760 revealed an AUC of 0.792(95% CI: 0.719–0.865), with 83.3% sensitivity and 69.1% specificity. (D, E, F) MiR-601 yielded an AUC of 0.638(95% CI: 0.530–0.747) with 72.1% sensitivity and 51.7% specificity(cut-off value = −10.73), and miR-760 revealed an AUC of 0.682(95% CI: 0.576–0.789) with 69.8% sensitivity and 62.1% specificity(cut-off value = −7.63) for discriminating advanced adenomas from controls. Combined analysis of miR-601 and miR-760 did not improve the diagnostic efficiency, and gave a similar AUC of 0.683(95% CI: 0.576–0.789) with 72.1% sensitivity and 62.1% specificity as miR-760.

To identify the 17 differentially expressed plasma miRNAs identified by expression profiling, their levels were measured by qRT-PCR in 90 CRCs and 58 normal controls normalized to spiked in cel-miR-39. Both miR-601 and miR-760 were significantly decreased in CRC plasma compared with the controls (P<0.0001, Figure 1). ROC curve analysis indicated their potential diagnostic value and the AUCs for miR-601 and miR-760 were 0.747(95% CI: 0.666–0.828) and 0.788(95% CI: 0.714–0.862), respectively. At a threshold of -11.50 for miR-601, the optimal sensitivity and specificity were 69.2% and 72.4% in separating CRC from normal controls. At a threshold of −8.09 for miR-760, the sensitivity and the specificity were 80.0% and 72.4%, respectively. Simultaneous analysis of miR-601 and miR-760 showed a similar AUC of 0.792 (95% CI: 0.719–0.865) as miR-760, with 83.3% sensitivity and 69.1% specificity, indicating a poor additive effect of the 2 miRNAs (Figure 2). We previously identified that plasma miR-29a and miR-92a have significant diagnostic value for CRC [15]. For most cases in that study was the same as that in this paper, these two targets were added for combined ROC curve analysis with miR-760. The resulting AUC increased to 0.943(95% CI: 0.908–0.979) with 83.3% sensitivity and 93.1% specificity in discriminating CRC from the control, suggesting the additive effect in the diagnostic value of these 3 miRNAs(Figure S1, S2). Addition of miR-601 to these three miRNAs did not improve their differentiation power between CRC patients and normal controls and resulted in a same AUC of 0.943(95% CI: 0.907–0.978). Additionally, no significant difference was found between CRC patients and controls in the remaining 15 miRNAs (*P*>0.05, data not shown). When the qRT-PCR data was normalized to miR-16 [15], plasma miR-601 and miR-760 were still significantly down-regulated in CRC when compared with normal controls (*P* = 0.002 for miR-601 and *P*<0.0001 for miR-760, Figure S3).

### Diagnostic Value of miR-601 and miR-760 for Advanced Adenomas

To continue our investigation of the diagnostic value of miR-601 and miR-760 in CRC development, we measured their plasma expression in 43 advanced adenomas, which were defined as precancerous lesions of CRC. Plasma levels of miR-601 and miR-760 were remarkably reduced in advanced adenomas compared with normal controls (*P* = 0.018 for both miR-601 and miR-760, Figure 1). Both the 2 miRNAs could weakly differentiate advanced adenomas from normal controls as shown by ROC curve analysis; and the AUC was 0.638 (95% CI: 0.530–0.747) for miR-601 and 0.682 (95% CI: 0.576–0.789) for miR-760. The optimal sensitivity and specificity of miR-601 were 72.1% and 51.7% at a cut-off value of −10.73; these values for miR-760 were 69.8% and 62.1% at a cut-off value of −7.63. Combined analysis of miR-601 and miR-760 failed to improve the diagnostic efficiency of miR-760 and showed a similar AUC of 0.683 (95% CI: 0.576–0.789), with 72.1% sensitivity and 62.1% specificity (Figure 2). Furthermore, expression of plasma miR-601 and miR-760 in advanced adenomas was significantly increased compared to CRCs (*P* = 0.0174 for miR-601, *P* = 0.0214 for miR-760, Figure 1). When normalized to miR-16, plasma miR-601 and miR-760 remained down-regulated in advanced adenomas compared with normal controls (*P* = 0.0198 for miR-601 and *P* = 0.0184 for miR-760, Figure S3).

### Association with Clinical Characteristics

**Figure 3:**
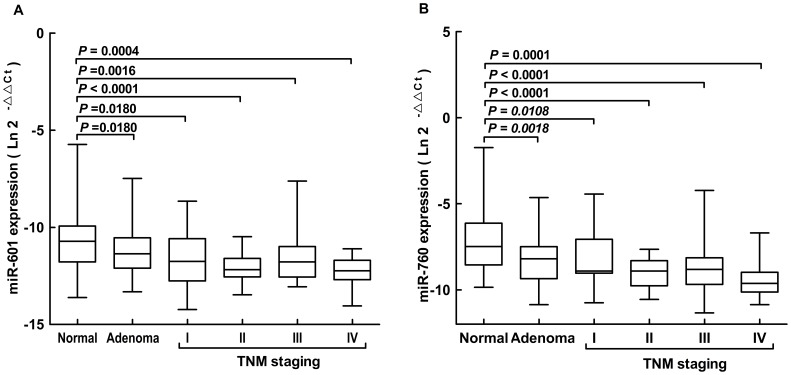
Association of plasma miR-601 and miR-760 expression with different TNM stages. Box plots of plasma levels of miR-601(A) and miR-760(B) in normal subjects (n = 58), advanced adenomas(n = 43) and CRC patients(n = 90) with different TNM stage (26 with I, 25 with II, 29 with III and 10 with IV). Expression levels of the miRNAs are normalized to cel-miR-39. The lines inside the boxes denote the medians. Mann-Whitney U test was used to determine statistical significance.

To demonstrate the decreased trend of plasma miR-601 and miR-760 expression in CRC development, CRC cases were further stratified by their TNM staging. The patients were separated into 5 groups, from advanced adenoma (the least advanced lesion) to Stage IV CRC(the most advanced lesion). Compared with normal controls, levels of plasma miR-601 and miR-760 were reduced in all the 5 groups. More importantly, this was in line with the down-regulation trend of the levels of the 2 miRNAs being lowest in stage IV patients and highest in stage I patients. Both the plasma levels of miR-601 and miR-760 were reduced during progression of CRC from normal through advanced adenoma to CRC (Figure 3). There was no significant association between these 2 miRNAs and gender, age, nodal status, tumor location, tumor size or histology (*P*>0.05, data not shown).

Carcinoembryonic antigen(CEA) is a biomarker used for monitoring CRC recurrence and is not a valid diagnostic biomarker from a clinical standpoint for its low sensitivity and specificity. However, there is no ideal potential biomarkers for CRC screening to date and CEA is still served as a preliminary tumor indicator in health examination. To assess possible complementation of plasma miRNA and CEA for the early detection of CRCs, the CEA data were compared with miR-601 and miR-760 levels in 51 stage I and stage II patients. The cut-off values of miR-601 and miR-760 from ROC curve analysis were −11.50 and −8.09, respectively. The cut-off value for CEA was 5.0 ng/mL (ln 5 = 1.609). MiR-601 or miR-760 identified an additional 26 or 29 cases missed by CEA alone, showing greater power in differentiating early stage CRCs. Combined use of miR-601 and miR-760 could detected 30 CEA-missed cases in all. ROC curve analysis showed that combining of miR-601 and miR-760 could improve the diagnostic sensitivity of CEA from 29.4% to 80.4% with an AUC of 0.805 (95% CI: 0.457–0.683) (Figure S4).

### MiR-601 and miR-760 Expression in CRC Tissues

**Figure 4:**
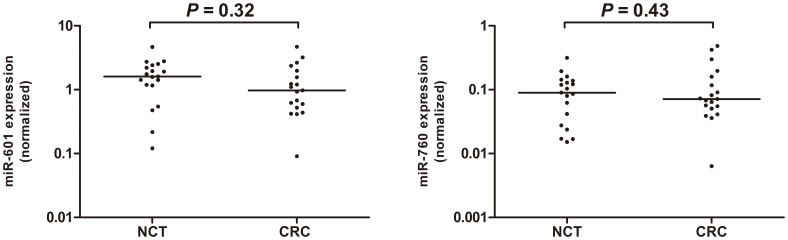
MiR-601 and miR-760 expression in CRC tissues. No significant differences about the miR-601(A) and miR-760(B) concentrations were found between CRC tissues and paired non-cancerous tissues (NCT) (n = 19). Expression levels of the miRNAs were normalized to RNU6B. The line represents the median value. Mann-Whitney U test was used to determine statistical significance.

For the purpose of digging out the inner relationship of miRNA expression between plasma and corresponding tumor tissue samples, miR-601 and miR-760 were measured in tumor tissues from another 19 CRC cases. No significant differences were found for both 2 miRNAs between cancer and noncancerous tissues (*P* = 0.32 for miR-601 and *P* = 0.43 for miR-760, Figure 4). The release of the miRNAs by the tumor cells into the circulation being affected by multiple parameters may explain the inconsistency. In addition, it is unknown whether tumor cells could change the miRNAs releasing into blood by other organs or cells. Larger samples validation was needed to confirm miR-601 and miR-760 expression in CRC tissues.

### MiR-601 and miR-760 Stability in Plasma

**Figure 5:**
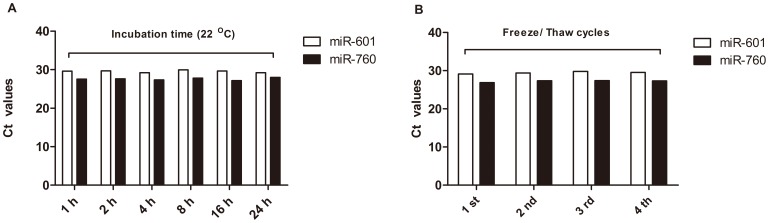
Plasma miR-601 and miR-760 stability experiment. Plasma levels of miR-601 and miR-760 were stable after prolonged room temperature incubation from 1 to 24 hours(A) or at least 4 freeze-thaw cycles(B).

Plasma levels of miR-601 and miR-760 were found to be stable after being subjected to prolonged incubation time at room temperature, and the Ct values didn’t show significant fluctuations. The similar results were found in the next freeze-thaw cycles experiment (Figure 5). Therefore, we considered that they were quite stable in plasma samples and hardly degraded during protocol and storage settings. Based on these results, we concluded that miR-601 and miR-760 basically met the requirements for clinical application.

## Discussion

MiRNAs play an important role in carcinogenesis. Deregulation of miRNA expression occurs in various cancers, notably miR-143 and miR-145 in CRC [23]. MiRNAs can contribute to therapeutic strategies as diagnostic biomarkers and prognostic factors in cancers [5], [6]. However, biopsy-based detecting methods remain invasive and time-consuming for the screening and monitoring of cancer patients. Tumor-derived miRNAs first described by Mitchell et al. clearly show that stable miRNAs are present in human plasma and serum, which opens up an extremely wide field for clinical application [9], [24], [25], [26], [27].

Published reports of circulating miRNAs in CRC are limited. However, Ng et al. found that miR-17-3p and miR-92 were significantly elevated in the serum of CRC patients with significant reduction following surgery. Further validation with an independent set of 180 samples indicated that miR-92 levels in plasma can distinguish between colorectal and gastric cancers, inflammatory bowel disease or normal subjects [13]. And we previously reported that plasma miR-29a and miR-92a were significantly up-regulated in CRC patients with good diagnostic values for CRC screening [15].

MiR-601 and miR-760 were 2 miRNAs of less concern in malignancies. MiR-601, located within *DENND1A* gene which up-regulated in uterine cervical cancer, could affect nuclear factor-kappa B signaling pathways in human lung cancer cells A549 [28], [29]. MiR-760, located within intron-1 of *BCAR3* gene, is regulated by estrogens and has a lower expression in CRC tissues [30]. No further functional studies have since being carried out on the 2 miRNAs. Information on their relationships with carcinogenesis remains limited, but our novel findings suggest that plasma miR-601 and miR-760 can act as suitable early diagnostic markers for CRC.

In this study, we firstly found miR-601 and miR-760 were down-regulated in CRC plasma by miRNA profiling and validated them by qRT-PCR from a total of 17 selected miRNAs. We demonstrated that the plasma levels of miR-601 and miR-760 were decreased in advanced colorectal neoplasia compared with healthy controls, and that miR-760 was more prominent than miR-601 as a CRC biomarker. According to our experience and previous studies, 11% concordance between chip screening and validation was low but acceptable. ROC curve analysis showed weak expression of these 2 miRNAs could contribute to the early detection of CRC with 69.2% sensitivity and 72.4% specificity for miR-601 (AUC = 0.747) and 80.0% sensitivity and 72.4% specificity for miR-760 (AUC = 0.788). The combination of these biomarkers complemented CEA in detecting early stage CRCs (stages I and II) got an AUC of 0.805 with 80.4% sensitivity and 65.5% specificity. Furthermore, combined miR-601 and miR-760 could also discriminate advanced adenomas from healthy controls giving an AUC of 0.683 with 72.1% sensitivity and 62.1% specificity. More remarkably, the levels of both miR-601 and miR-760 fell down in stages I and II CRC, which strongly elevated their potential value for the early detection of CRC. Although the diagnostic value of miR-601 and miR-760 may fall short of being optimal, a panel of three plasma miRNA (miR-760, -29a and -92a) markers revealed good diagnostic efficiency.

No consensus internal controls that are crucially important in accurate quantification of RNA levels with qRT-PCR have been established to date. It was confirmed that a linear correlation was existed between the amount of inputted synthetic miRNA and the Ct values in qRT-PCR [31]. In this study, cel-miR-39 was added to each denatured sample during the RNA isolation procedures for normalization [32]. In addition, we also used miR-16, a suggested internal control by our previous work, to normalize our qRT-PCR data and obtained similar results (Figure S3) [15]. However, it seemed that we can get a better AUC when normalizing with cel-miR-39, so cel-miR-39 was selected to normalize the qRT-PCR data in this study (Figure S5).

Plasma samples can be collected non-invasively and are not time-limited. We have established that circulating miRNAs extracted from a minimal plasma sample were well suited for quantification purposes. In comparison to other nucleotide-containing molecules, such as DNA and mRNA, miRNAs are more resistant to DNase and RNase activity, making them relatively stable in the circulation. We proved that miR-601 and miR-760 were quite stable in plasma samples and underwent little degradation after prolonged room temperature incubation or several freeze-thaw cycles.

One limitation of this study has been that molecular insights into the cause of the deregulation have not been forthcoming. Circulation miRNA levels appear to reflect tissue expression which has been shown in several cancer types including prostate cancer and breast cancer [9], [24]. While miR-601 and miR-760 levels were not decreased in CRC tissues, which seemed not consist with that in plasma. Another limitation of this study is that similar, but not complementary, diagnostic values were found between these two miRNAs. Addition of miR-601 did not improve the differential power of miR-760 in discriminating advanced colorectal neoplasia from normal controls. However, identifying plasma miR-601 and miR-760 as biomarkers should now more accurately detect CRC in its early stage. Further more, addition of miR-760 to others two miRNA identified previously by us revealed a significant improved diagnostic value, suggesting that a panel of plasma miRNA markers may improve the sensitivity and specificity of this assay for CRC screening.

In conclusion, plasma miR-601 and miR-760 levels in CRC and advanced adenomas are significantly decreased, suggesting the possibility that plasma miR-601 and miR-760 are novel biomarkers for the clinic diagnosis of CRC, but underlying mechanisms of their decrease require further investigation.

## Supporting Information

Figure S1 **Plasma miR-29a and miR-92a expression.** Plasma miR-29a(A) and miR-92a(B) were significantly up-regulated in CRCs compared with normal controls (*P*<0.0001, *P* = 0.0005). Adenomas were simultaneously differentiated from normal controls (*P* = 0.0015, *P* = 0.0003). The line represents the median value. Mann-Whitney U test was used to determine statistical significance.
(TIF)

Figure S2 **Combined ROC curve analysis of miR-29a, miR-92a and miR-760.** Combined ROC curve analysis of the 3 miRNAs(miR-29a, miR-92a and miR-760) yielded an AUC of 0.943 (95% CI: 0.908–0.979)with 83.3% sensitivity and 93.1% specificity in discriminating CRC from normal controls.
(JPG)

Figure S3 **Plasma miR-601 and miR-760 expression normalized by miR-16.** Both cel-miR-39 and miR-16 could be used for normalization. MiR-601 and miR-760 were still significantly down regulated in CRC and advanced adenomas compared with normal controls when the qRT-PCR data was normalized to miR-16. The line represents the median value. Mann-Whitney U test was used to determine statistical significance.
(TIF)

Figure S4 **Diagnostic sensitivities of miR-601 and miR-760 compared with CEA for stage I and II CRCs.** The cut-off values of miR-601 and miR-760 from ROC curve analysis were -11.50 and -8.09, respectively. The cut-off value for CEA was 5.0 ng/mL (ln 5 = 1.609). (A, B) Two-parameter classifications showed that 26 and 29 cases (stage I and II) missed by CEA were supplementary detected by miR-601 and miR-760, respectively. (C) MiR-601 and miR-760 were more potent than CEA in differentiating stage I and II CRCs from healthy controls and combined use of miR-601 and miR-760 could detected 30 CEA-missed cases in all. (D) Combined use of the 3 markers yielded an AUC of 0.805(95% CI: 0.457–0.683) with an elevated sensitivity of 86.3%.
(TIF)

Figure S5 **ROC curve analysis of plasma miR-601 and miR-760 normalized to cel-miR-39 or miR-16.** (A, B, C) Normalized to cel-miR-39, plasma miR-601 yield an AUC of 0.713(95% CI: 0.634–0.792)with 72.4% sensitivity and 62.4% specificity(cut-off value = −11.50), and miR-760 yield an AUC of 0.754 (95% CI: 0.682–0.826)with 72.4% sensitivity and 72.2% specificity(cut-off value = −8.08) for discriminating advanced colorectal neoplasia (CRC and advanced adenomas) from normal controls. Combined analysis of miR-601 and miR-760 revealed an AUC of 0.824 (95% CI: 0.680–0824), with 74.4% sensitivity and 70.0% specificity. (D, E, F) Normalized to miR-16, plasma miR-601 yielded an AUC of 0.668(95% CI: 0.588–0.748) with 74.1% sensitivity and 53.4% specificity(cut-off value = −7.63), and miR-760 revealed an AUC of 0.724(95% CI: 0.651–0.798) with 87.9% sensitivity and 48.9% specificity(cut-off value = −4.81) for discriminating advanced colorectal neoplasia from normal controls. Combined analysis of miR-601 and miR-760 revealed an AUC of 0.732(95% CI: 0.661–0.803), with 43.6% sensitivity and 94.8% specificity.
(JPG)

Table S1 **Patient information for miRNA profiling.**
(DOCX)

Table S2 **Patient information for large scale validation.**
(DOC)

Table S3 **The pathological features of advanced adenomas.**
(DOCX)

Table S4 **Patient information for validation of paired CRC tissues.**
(DOCX)

Table S5 **miRNAs deregulated in CRC plasma with FC>2.**
(DOCX)
