# Supplementary figures and images for: Plasma miR-601 and miR-760 Are Novel Biomarkers for the Early Detection of Colorectal Cancer
Source: PLoS One. 2012 Sep 6;7(9):e44398. doi: 10.1371/journal.pone.0044398 (PMC3435315; doi:10.1371/journal.pone.0044398)

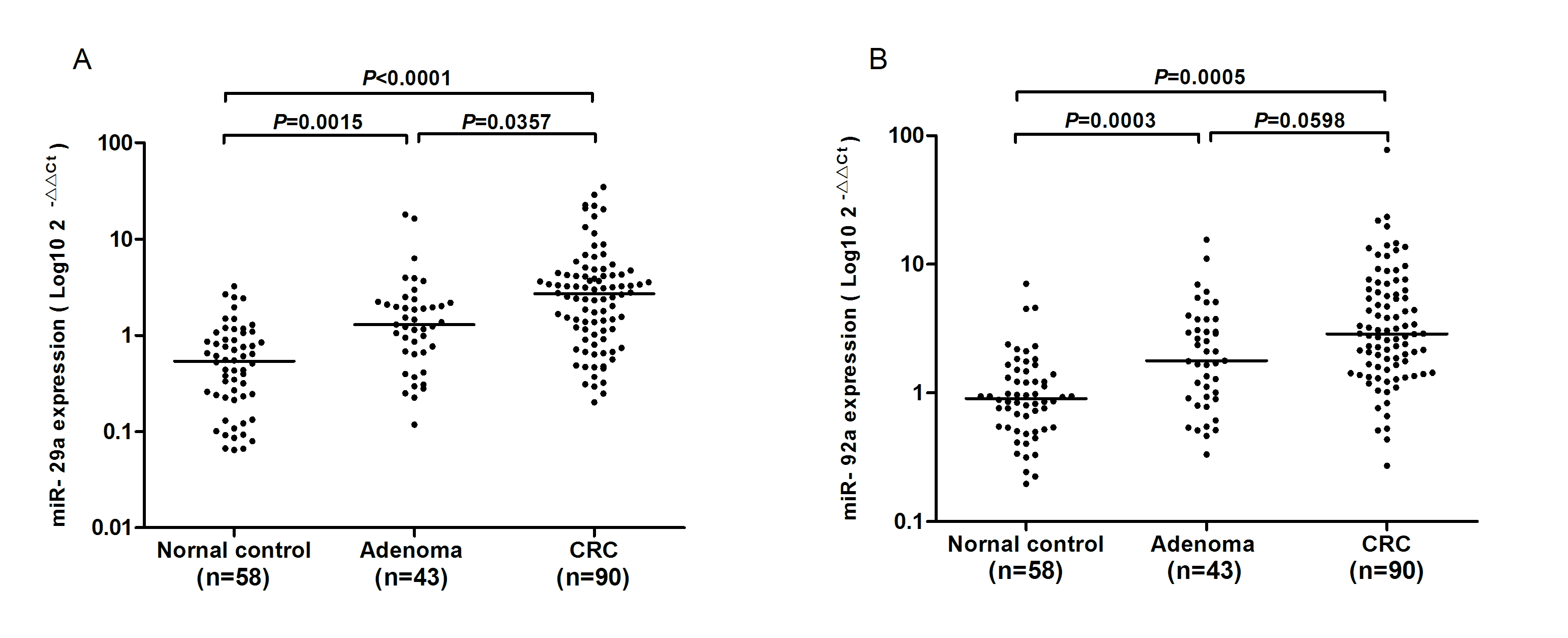

Supplement: Figure S1 — Plasma miR-29a and miR-92a expression. Plasma miR-29a(A) and miR-92a(B) were significantly up-regulated in CRCs compared with normal controls (P<0.0001, P = 0.0005). Adenomas were simultaneously differentiated from normal controls (P = 0.0015, P = 0.0003). The line represents the median value. Mann-Whitney U test was used to determine statistical significance. (TIF) [file pone.0044398.s001.tif]

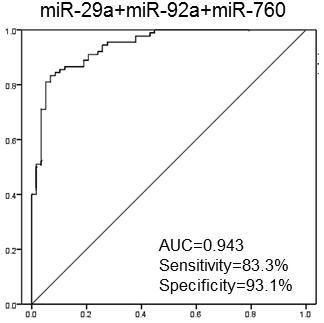

Supplement: Figure S2 — Combined ROC curve analysis of miR-29a, miR-92a and miR-760. Combined ROC curve analysis of the 3 miRNAs(miR-29a, miR-92a and miR-760) yielded an AUC of 0.943 (95% CI: 0.908–0.979)with 83.3% sensitivity and 93.1% specificity in discriminating CRC from normal controls. (JPG) [file pone.0044398.s002.jpg]

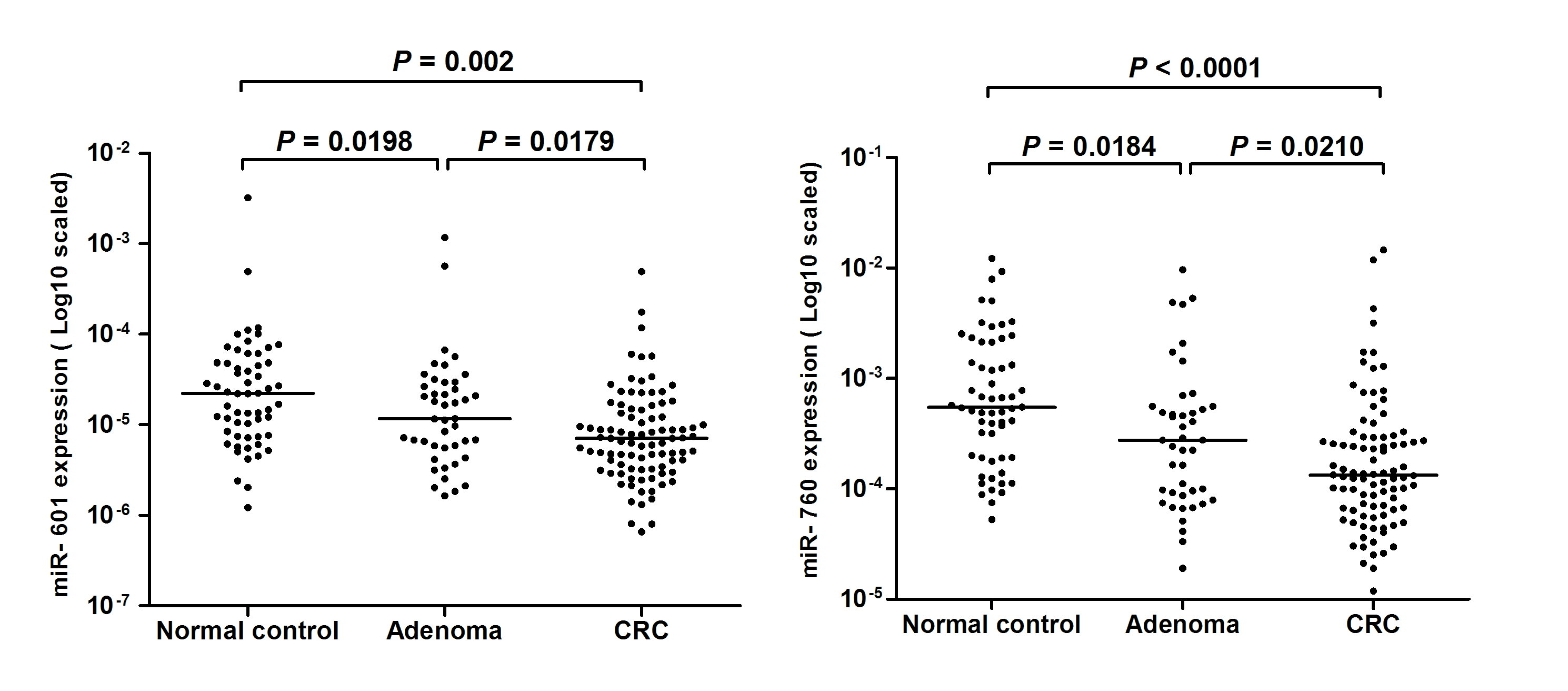

Supplement: Figure S3 — Plasma miR-601 and miR-760 expression normalized by miR-16. Both cel-miR-39 and miR-16 could be used for normalization. MiR-601 and miR-760 were still significantly down regulated in CRC and advanced adenomas compared with normal controls when the qRT-PCR data was normalized to miR-16. The line represents the median value. Mann-Whitney U test was used to determine statistical significance. (TIF) [file pone.0044398.s003.tif]

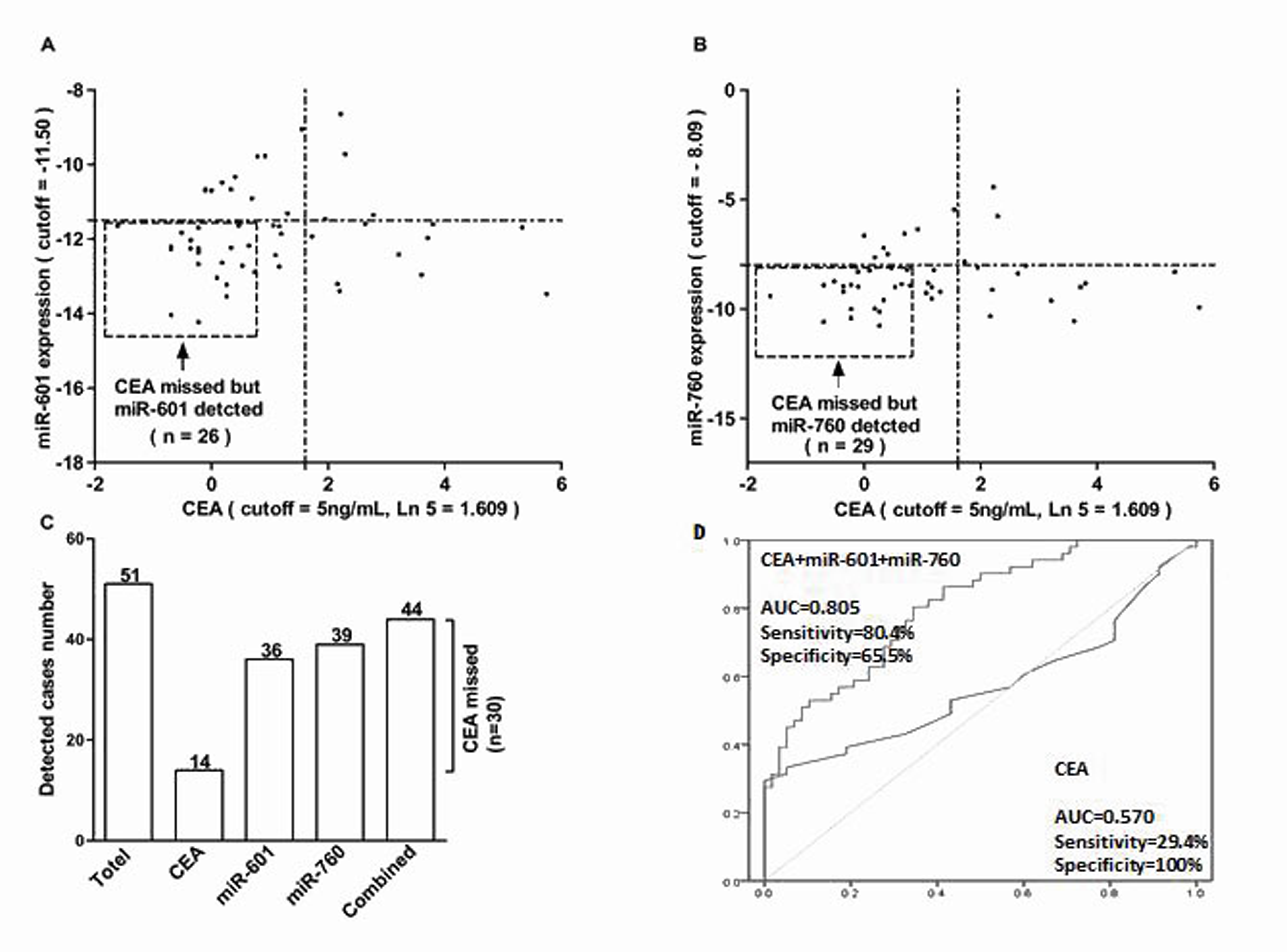

Supplement: Figure S4 — Diagnostic sensitivities of miR-601 and miR-760 compared with CEA for stage I and II CRCs. The cut-off values of miR-601 and miR-760 from ROC curve analysis were -11.50 and -8.09, respectively. The cut-off value for CEA was 5.0 ng/mL (ln 5 = 1.609). (A, B) Two-parameter classifications showed that 26 and 29 cases (stage I and II) missed by CEA were supplementary detected by miR-601 and miR-760, respectively. (C) MiR-601 and miR-760 were more potent than CEA in differentiating stage I and II CRCs from healthy controls and combined use of miR-601 and miR-760 could detected 30 CEA-missed cases in all. (D) Combined use of the 3 markers yielded an AUC of 0.805(95% CI: 0.457–0.683) with an elevated sensitivity of 86.3%. (TIF) [file pone.0044398.s004.tif]

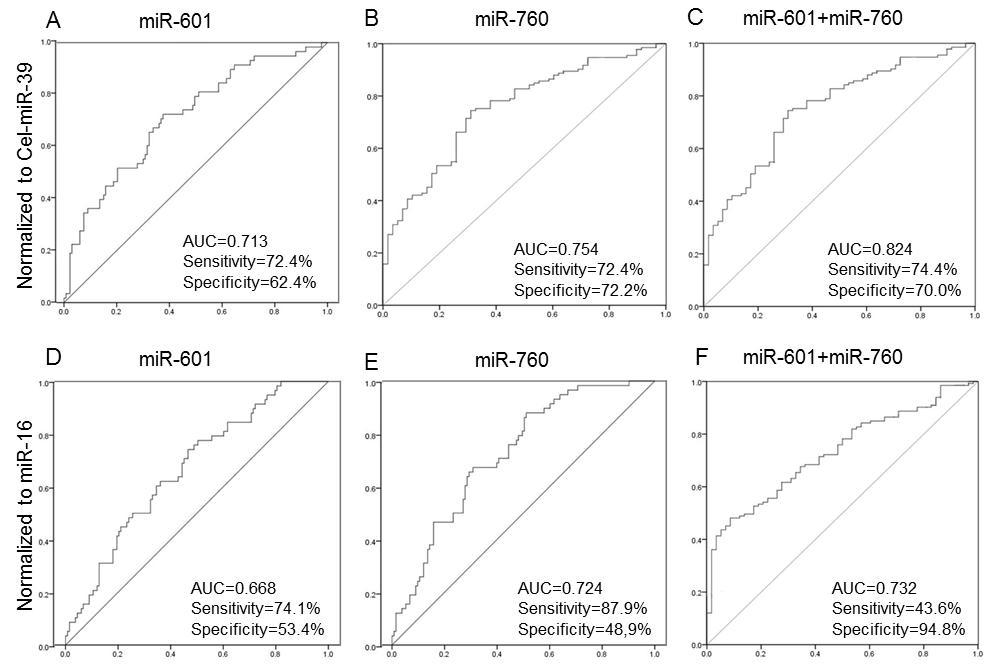

Supplement: Figure S5 — ROC curve analysis of plasma miR-601 and miR-760 normalized to cel-miR-39 or miR-16. (A, B, C) Normalized to cel-miR-39, plasma miR-601 yield an AUC of 0.713(95% CI: 0.634–0.792)with 72.4% sensitivity and 62.4% specificity(cut-off value = −11.50), and miR-760 yield an AUC of 0.754 (95% CI: 0.682–0.826)with 72.4% sensitivity and 72.2% specificity(cut-off value = −8.08) for discriminating advanced colorectal neoplasia (CRC and advanced adenomas) from normal controls. Combined analysis of miR-601 and miR-760 revealed an AUC of 0.824 (95% CI: 0.680–0824), with 74.4% sensitivity and 70.0% specificity. (D, E, F) Normalized to miR-16, plasma miR-601 yielded an AUC of 0.668(95% CI: 0.588–0.748) with 74.1% sensitivity and 53.4% specificity(cut-off value = −7.63), and miR-760 revealed an AUC of 0.724(95% CI: 0.651–0.798) with 87.9% sensitivity and 48.9% specificity(cut-off value = −4.81) for discriminating advanced colorectal neoplasia from normal controls. Combined analysis of miR-601 and miR-760 revealed an AUC of 0.732(95% CI: 0.661–0.803), with 43.6% sensitivity and 94.8% specificity. (JPG) [file pone.0044398.s005.jpg]
