# Supplementary material for: Plasma miR-601 and miR-760 Are Novel Biomarkers for the Early Detection of Colorectal Cancer
Source: PLoS One. 2012 Sep 6;7(9):e44398. doi: 10.1371/journal.pone.0044398 (PMC3435315; doi:10.1371/journal.pone.0044398)
Supplement: Table S1 — Patient information for miRNA profiling. (DOCX) [file pone.0044398.s006.docx]

**Table S1. Patient information for miRNA profiling.**

| Characteristics | Colorectal cancer (n=10) | Normal control (n=10) |
| --- | --- | --- |
| Gender |  |  |
| Male | 5 | 5 |
| Female | 5 | 5 |
| Age |  |  |
| Mean(SD) | 60 (8) | 58（10） |
| Median(range) | 60（45-80） | 56（39-77） |
| TMN stage |  |  |
| Ⅱ | 5 |  |
| Ⅲ | 5 |  |
| T stage |  |  |
| 2 | 3 |  |
| 3 | 7 |  |
| Nodal status |  |  |
| Positive | 5 |  |
| Negative | 5 |  |
| Tumor lacation |  |  |
| Rectum | 2 |  |
| Distal colon | 3 |  |
| Proximal colon | 5 |  |
| Histological |  |  |
| Adenocarcinoma | 10 |  |
