# Supplementary material for: Plasma miR-601 and miR-760 Are Novel Biomarkers for the Early Detection of Colorectal Cancer
Source: PLoS One. 2012 Sep 6;7(9):e44398. doi: 10.1371/journal.pone.0044398 (PMC3435315; doi:10.1371/journal.pone.0044398)
Supplement: Table S2 — Patient information for large scale validation. (DOC) [file pone.0044398.s007.doc]

**Table S2. Patient information for large scale validation.**

| **Characteristics** | **Colorectal cancer n=90** | **Advanced adenomas n=43** | **Normal control n=58** |
| --- | --- | --- | --- |
| **Gender** |  |  |  |
| Male | 45 | 23 | 30 |
| Female | 45 | 20 | 28 |
| **Age** |  |  |  |
| Mean(SD) | 62 (11) | 55（11） | 58（12） |
| Median(range) | 61（21-84） | 53（33-77） | 56（36-85） |
| **TMN stage** |  |  |  |
| Ⅰ | 26 |  |  |
| Ⅱ | 25 |  |  |
| Ⅲ | 29 |  |  |
| Ⅳ | 10 |  |  |
| **T stage** |  |  |  |
| 1 | 4 |  |  |
| 2 | 20 |  |  |
| 3 | 37 |  |  |
| 4 | 29 |  |  |
| **Nodal status** |  |  |  |
| Positive | 66 |  |  |
| Negative | 24 |  |  |
| **Tumor lacation** |  |  |  |
| Rectum | 48 | 23 |  |
| Distal colon | 19 | 11 |  |
| Proximal colon | 23 | 8 |  |
| **Histological** |  |  |  |
| Adenocarcinoma | 80 |  |  |
| Mucious adenocarcinoma | 9 |  |  |
| Signet ring cell | 1 |  |  |
