# Supplementary material for: Plasma miR-601 and miR-760 Are Novel Biomarkers for the Early Detection of Colorectal Cancer
Source: PLoS One. 2012 Sep 6;7(9):e44398. doi: 10.1371/journal.pone.0044398 (PMC3435315; doi:10.1371/journal.pone.0044398)
Supplement: Table S3 — The pathological features of advanced adenomas. (DOCX) [file pone.0044398.s008.docx]

**Table S3.The pathological features of advanced adenomas.**

| Diameter≥1 cm | + |  |  | + | + |  | + |
| --- | --- | --- | --- | --- | --- | --- | --- |
| Villous components |  | + |  | + |  | + | + |
| High-grade dysplasia |  |  | + |  | + | + | + |
| Number | 23 | 24 | 29 | 13 | 15 | 19 | 13 |
