# Supplementary material for: Plasma miR-601 and miR-760 Are Novel Biomarkers for the Early Detection of Colorectal Cancer
Source: PLoS One. 2012 Sep 6;7(9):e44398. doi: 10.1371/journal.pone.0044398 (PMC3435315; doi:10.1371/journal.pone.0044398)
Supplement: Table S4 — Patient information for validation of paired CRC tissues. (DOCX) [file pone.0044398.s009.docx]

**Table S4. Patient information for validation of paired CRC tissues.**

| Characteristics | Paired CRC and non-cancerous tissues n=19 |
| --- | --- |
| Gender |  |
| Male | 11 |
| Female | 8 |
| Age |  |
| Mean(SD) | 70 (9) |
| Median(range) | 68（49-79） |
| TMN stage |  |
| Ⅱ | 7 |
| Ⅲ | 12 |
| T stage |  |
| 2 | 6 |
| 3 | 13 |
| Nodal status |  |
| Positive | 7 |
| Negative | 12 |
| Tumor lacation |  |
| Rectum | 3 |
| Distal colon | 6 |
| Proximal colon | 10 |
| Histological |  |
| Adenocarcinoma | 19 |
