# Supplementary material for: Plasma miR-601 and miR-760 Are Novel Biomarkers for the Early Detection of Colorectal Cancer
Source: PLoS One. 2012 Sep 6;7(9):e44398. doi: 10.1371/journal.pone.0044398 (PMC3435315; doi:10.1371/journal.pone.0044398)
Supplement: Table S5 — miRNAs deregulated in CRC plasma with FC>2. (DOCX) [file pone.0044398.s010.docx]

**Table S5.miRNAs deregulated in CRC plasma with FC>2.**

| miRNA Name | Fold change(>2) | Up/Down regulation |
| --- | --- | --- |
| hsa-miR-125a-5p | 74.84 | Up |
| hsa-let-7e | 10.60 | Up |
| hsa-miR-24 | 7.27 | Up |
| has-miR-210 | 7.11 | Up |
| hsa-miR-22* | 6.99 | Up |
| hsa-miR-19a | 6.97 | Up |
| hsa-miR-221 | 6.63 | Up |
| hsa-miR-376a | 6.56 | Up |
| hsa-miR-92a | 5.34 | Up |
| hsa-miR-590-5p | 4.66 | Up |
| hsa-miR-142-5p | 3.96 | Up |
| hsa-miR-30a | 3.77 | Up |
| hsa-miR-135b | 3.73 | Up |
| hsa-miR-151-5p | 3.54 | Up |
| hsa-miR-34b* | 3.54 | Up |
| hsa-miR-423-3p | 3.50 | Up |
| hsa-miR-146a | 3.25 | Up |
| hsa-miR-652 | 3.23 | Up |
| hsa-miR-199a-5p | 3.15 | Up |
| hsa-miR-124 | 3.10 | Up |
| hsa-miR-650 | 2.99 | Up |
| hsa-miR-29a | 2.96 | Up |
| hsa-miR-361-3p | 2.90 | Up |
| hsa-miR-106b* | 2.86 | Up |
| hsa-miR-135a | 2.83 | Up |
| hsa-miR-375 | 2.82 | Up |
| hsa-miR-95 | 2.64 | Up |
| hsa-miR-409-3p | 2.62 | Up |
| hsa-miR-339-3p | 2.58 | Up |
| hsa-miR-28-3p | 2.54 | Up |
| hsa-miR-200c | 2.50 | Up |
| hsa-miR-148a | 2.49 | Up |
| hsa-miR-191 | 2.39 | Up |
| hsa-miR-183 | 2.35 | Up |
| hsa-miR-18b | 2.34 | Up |
| hsa-miR-199a-3p | 2.33 | Up |
| hsa-miR-502-3p | 2.28 | Up |
| hsa-miR-20a | 2.26 | Up |
| hsa-miR-29c | 2.25 | Up |
| hsa-miR-628-3p | 2.25 | Up |
| hsa-miR-200a | 2.18 | Up |
| hsa-miR-205 | 2.16 | Up |
| hsa-miR-181b | 2.13 | Up |
| hsa-miR-29b | 2.10 | Up |
| hsa-let-7c | 0.50 | Down |
| hsa-miR-532-5p | 0.50 | Down |
| hsa-miR-564 | 0.48 | Down |
| hsa-miR-1260 | 0.48 | Down |
| hsa-miR-342-3p | 0.47 | Down |
| hsa-miR-128 | 0.45 | Down |
| hsa-let-7d | 0.44 | Down |
| hsa-let-7f-1* | 0.44 | Down |
| hsa-miR-144* | 0.44 | Down |
| hsa-miR-502-5p | 0.40 | Down |
| hsa-miR-501-3p | 0.39 | Down |
| hsa-miR-301b | 0.38 | Down |
| hsa-miR-1974 | 0.36 | Down |
| hsa-miR-142-3p | 0.35 | Down |
| hsa-miR-934 | 0.34 | Down |
| hsa-miR-331-3p | 0.34 | Down |
| hsa-miR-143 | 0.32 | Down |
| hsa-miR-627 | 0.31 | Down |
| hsa-miR-34a | 0.31 | Down |
| hsa-miR-491-5p | 0.30 | Down |
| hsa-miR-145 | 0.30 | Down |
| hsa-miR-605 | 0.29 | Down |
| hsa-miR-1909 | 0.28 | Down |
| hsa-miR-346 | 0.27 | Down |
| hsa-miR-520h | 0.26 | Down |
| hsa-miR-615-3p | 0.23 | Down |
| hsa-miR-1224-3p | 0.21 | Down |
| hsa-miR-454 | 0.21 | Down |
| hsa-miR-329 | 0.21 | Down |
| hsa-miR-141 | 0.16 | Down |
| hsa-miR-150 | 0.16 | Down |
| hsa-miR-188-3p | 0.16 | Down |
| hsa-miR-192 | 0.15 | Down |
| hsa-miR-10a | 0.14 | Down |
| hsa-miR-572 | 0.14 | Down |
| hsa-miR-425* | 0.14 | Down |
| hsa-let-7a | 0.13 | Down |
| hsa-miR-224* | 0.12 | Down |
| hsa-miR-720 | 0.10 | Down |
| hsa-miR-601 | 0.09 | Down |
| hsa-miR-495 | 0.06 | Down |
| hsa-miR-760 | 0.04 | Down |
